# Differences in Dementia Diagnosis Rates in Primary Care in Traditional Medicare and Medicare Advantage

**DOI:** 10.1002/alz.089315

**Published:** 2025-01-09

**Authors:** Sidra Haye, Mireille Jacobson, Julie M Zissimopoulos

**Affiliations:** ^1^ University of Southern California, Los Angeles, CA USA

## Abstract

**Background:**

Approximately half of Medicare beneficiaries enroll in Medicare Advantage (MA) plans. Unlike Traditional Medicare (TM), MA plans coordinate patient care through physician networks and care practices, often relying on Primary Care Providers (PCPs). PCPs also play an important role in diagnosing dementia; the majority of persons living with dementia are diagnosed by PCPs. Studies document that dementia diagnosis rates are lower in MA than TM. There is a gap in knowledge about how barriers to and incentives for diagnosing dementia among physicians in MA and TM impact rates of diagnosis. To examine whether incident dementia diagnosis rates vary for beneficiaries in TM and MA seen by the same Primary Care Provider (PCP).

**Methods:**

We use propensity score matching and linear regression models, with and without PCP fixed effects, to analyze differences in incident dementia diagnoses in 2017 in MA relative to TM. The analysis includes 100% of Medicare claims for TM and MA beneficiaries who were 65 and over and who were continuously enrolled in Part D and in either TM or MA from 2016 to 2018 or died in 2018. Each beneficiary is assigned to the PCP with whom they have the maximum number of evaluation and management visits in a year.

**Results:**

Among 15,633,290 beneficiaries with assigned PCPs, the dementia diagnosis rate was 3.12% in TM and 2.78% in MA. Adjusting for patient characteristics, we find that the probability of incident dementia diagnosis was 0.27 percentage points lower for MA patients compared to TM. Adjusting for patient characteristics and PCP, we find that the probability of incident dementia diagnosis was 0.12 percentage points lower (around 4% lower relative to TM mean) for MA patients compared to TM patients assigned to the same PCP.

**Conclusion:**

We find that even for a matched sample of TM and MA patients with the same PCP, dementiadiagnosis rates are lower in MA relative to TM. Differences in care practices as indicated by access to dementia specialists and incentives for coding dementia diagnosis may contribute to some of the differences in diagnosis rates.

